# Navigating narcolepsy: exploring coping strategies and their association with quality of life in patients with narcolepsy type 1

**DOI:** 10.1038/s41598-024-62698-5

**Published:** 2024-05-23

**Authors:** Giorgia Varallo, Christian Franceschini, Giada Rapelli, Corrado Zenesini, Valentina Baldini, Flavia Baccari, Elena Antelmi, Fabio Pizza, Luca Vignatelli, Francesco Biscarini, Francesca Ingravallo, Giuseppe Plazzi

**Affiliations:** 1 Department of Biomedical, Metabolic and Neural Sciences, University of Modena and Reggio Emilia, Modena, Italy; 2 Department of Medicine and Surgery, University of Parma, Parma, Italy; 3 IRCCS Istituto Delle Scienze Neurologiche Di Bologna, Bologna, Italy; 4 Department of Biomedical and Neuromotor Sciences (DIBINEM), Alma Mater Studiorum, University of Bologna, Bologna, Italy; 5 Department of Neurosciences, Biomedicine and Movement Sciences, University of Verona, Verona, Italy; 6 Department of Medical and Surgical Sciences (DIMEC), Alma Mater Studiorum, University of Bologna, Bologna, Italy

**Keywords:** Neuroscience, Psychology, Neurology

## Abstract

Narcolepsy type 1 (NT1) is a chronic neurological disorder characterized by symptoms such as excessive daytime sleepiness, sudden sleep episodes, disrupted nocturnal sleep, cataplexy, sleep paralysis, and hypnagogic hallucinations, which significantly impact the overall well-being and quality of life of individuals. While psychological factors have gained attention, there is limited research on the coping strategies employed by patients with NT1 and their association with quality of life. This study aimed to compare coping strategies in patients with NT1 and controls, as well as assess the relationship between coping strategies and quality of life in patients with NT1. A total of 122 individuals diagnosed with NT1 and 138 controls were enrolled in this cross-sectional study. Participants completed questionnaires assessing coping strategies and health-related quality of life. A Mann–Whitney U test was conducted to compare the use of different coping strategies by patients with NT1 and controls. Spearman's rho correlation was performed to examine the association between coping strategies and quality of life in the NT1 group. Results showed that patients with NT1 exhibited differences in the use of coping strategies compared to controls. They reported lower use of active coping, planning, instrumental, and emotional social support, and higher use of behavioral and mental disengagement. Denial and behavioral disengagement were significantly and negatively associated with quality of life. Identifying coping strategies and their association with quality of life may aid in the development of tailored interventions aimed at improving the adoption of effective coping strategies and reducing the use of maladaptive coping strategies.

## Introduction

Narcolepsy type 1 (NT1) is a neurological disorder that presents a range of symptoms, including excessive daytime sleepiness, sudden sleep episodes, cataplexy, disrupted nocturnal sleep, sleep paralysis, and hypnagogic hallucinations^1^, all of which significantly impair various aspects of life, encompassing physical, mental, and social domains of health^2–4^. Cataplexy stands as the pathognomonic clinical manifestation of NT1 and is defined as an abrupt occurrence of involuntary muscle atonia or paralysis that transpires during a state of wakefulness. It is mainly triggered by intense emotional stimuli, and its duration ranges from several seconds to several minutes. People with NT1 encounter significant barriers to actively functioning in their jobs, school, and social relationships^4,5^ due to the presence of cataplexy and excessive daytime sleepiness.

Managing NT1 requires a combination of pharmacological and non-pharmacological approaches to effectively reduce symptoms and improve the quality of life. Pharmacological therapies are essential in the treatment of NT1 symptoms. First- line treatments encompass drugs based on grade A (i.e., randomized controlled trials) and FDA/EMA approval or other compounds whose prescriptions are based on expert‘s opinions and clinical experience and are differently effective on the constellation of NT1 symptoms, administered in monotherapy or in combination^6^.

Non-pharmacological approaches are also important and often used in conjunction with medication^5,7^. Lifestyle modifications, such as maintaining a regular sleep schedule, practicing good sleep hygiene, and incorporating planned naps into daily routines, are recommended behavioral strategies to manage excessive sleepiness^7^.

It comes as no surprise that individuals with narcolepsy frequently report low levels of quality of life^3,8^ due to this constellation of disrupting symptoms and the impact of illness on overall functioning^2,4,5,9,10^. The challenging and lifelong management of this condition and its distressing symptoms can be important sources of psychological suffering^11^. Indeed, anxiety and depression are common co-occurring disorders in people with narcolepsy and could further worsen the quality of life^11^.

While symptomatic management remains crucial, efforts should be directed toward addressing the multifaceted impact of narcolepsy on physical, emotional, and social well-being. In recent years, increasing emphasis has been placed on the psychological aspects and their potential impact on overall health and quality of life^5,10,12–14^. However, one critical factor has received little attention: coping strategies, which can be defined as cognitive and behavioral efforts to manage the internal and external demands of a stressful situation^15,16^. Coping strategies are crucial to the management of chronic health conditions in promoting quality of life^17–19^ and have been extensively studied in several chronic health conditions^18^. In fact, living with a chronic disease such as NT1 requires significant adjustments and effective coping strategies for navigating the associated physical, emotional, and social challenges.

Coping strategies may be both adaptive and maladaptive. Adaptive coping strategies aid in the appropriate management and adjustment to the challenges given by chronic illnesses. Usually, they entail proactive engagement in problem-solving, such as acquiring information, focusing attention on the situation, and acceptance. For instance, a meta-analysis indicated that those who actively coped with diabetes by planning and gaining information had better glucose control and less anxiety and depression symptoms than those who did not^20^. On the other hand, maladaptive coping strategies are ineffective or harmful to general well-being. These could involve disengagement, denial, or engaging in unhealthy behaviors (e.g., alcohol or substance use) as a means of escape. The use of these coping strategies resulted associated with adverse physical and psychological outcomes in health conditions such as diabetes^21^, cancer^22^ and multiple sclerosis^23^.

As a result, it appears that studying coping strategies in NT1 is a critical area with an unfortunate paucity of evidence. Although NT1 has a significant impact on people's daily lives, it is still unclear which specific coping strategies are used by those who are affected. Understanding and evaluating coping strategies employed by patients with NT1 and their relationship with quality of life can help develop tailored non-pharmacological interventions aimed at promoting a better adaptation to illness. Therefore, the objective of this study was twofold: (i) to compare coping strategies among patients with NT1 and people without sleep disorders, and (ii) to evaluate the association between coping strategies and quality of life among patients with NT1.

## Methods

Cross-sectional study with a comparison group. The STROBE (Strengthening the Reporting of Observational Studies in Epidemiology)^24^ guidelines were followed. The “Psychosocial impact of narcolepsy” (cit D’Alterio) research study was conducted at the Narcolepsy Center of Bologna, Italy, over a period from February 2017 to July 2019. Patients were enrolled according to the following inclusion criteria: (i) age > 18; (ii) diagnosis of NT1 according to the International Classification of Sleep Disorders^25^; (iii) being able to understand the study purposes; (iv) reading written Italian. People without sleep disorders, matched by age and sex, were included in a comparison group (CG). The CG was recruited from family members or companions accompanying patients with narcolepsy or other neurological disorders (e.g., headache, neuromuscular disorders) at the tertiary neurological outpatient clinic of the IRCCS Institute of Neurological Sciences of Bologna. The study was approved by the Ethical Committee (protocol number: Comitato Etico Interaziendale Bologna-Imola, protocol number: 16181), and informed consent was obtained from all participants. The study has been performed in accordance with the Declaration of Helsinki's ethical principles.

### Measures

All participants underwent an ad hoc interview that collected sociodemographic information (such as sex, age in years, education and working status) and filled in validated questionnaires to investigate coping strategies and quality of life.

Coping strategies. The Coping Orientation to Problems Experienced Inventory (COPE) is a widely used questionnaire that measures coping strategies^26^. The COPE consists of 60 items that measure 15 different coping strategies. The 15 coping strategies are: active coping, planning, suppression of competing activities, restraint, use of instrumental social support, use of emotional social support, focusing on and venting of emotions, positive reinterpretation and growth, acceptance, religion, humor, denial, behavioral disengagement, mental disengagement, substance use. Descriptions of these coping strategies are provided in Supplementary Material Table S1. Respondents rate each item on a 4-point scale ranging from "I usually don't do this at all" to "I usually do this a lot".

Quality of life. The EQ-5D (EuroQol-5 Dimensions) is a standardized instrument used to assess health-related quality of life^27^. It consists of two parts: a descriptive system and a visual analogue scale (VAS). For the purpose of this study, we used only the descriptive system, which comprises five dimensions that cover different aspects of health: (i) Mobility: the ability to move around and perform daily activities; (ii) Self-Care: the ability to take care of oneself, such as bathing, dressing, or eating; (iii) Usual Activities: the ability to engage in usual activities, including work, study, housework, or leisure activities, (iv) Pain/Discomfort: the presence and severity of pain or discomfort; (v) Anxiety/Depression: the presence and severity of anxiety or depression symptoms. For each dimension, participants indicate their health status on a three-level scale: 1 = no problems, 2 = some problems, or 3 = extreme problems. A summary index with a maximum score of 1 can be derived from these five dimensions. The maximum score of 1 indicates the best health condition.

Finally, age at onset of symptoms, age at diagnosis, and current pharmacological therapy were all recorded for patients with NT1.

### Statistical analysis

For categorical variables, descriptive statistics were presented as absolute (n) and relative frequency (%), whereas for continuous variables, they were presented as mean with standard deviation (SD) and as median with interquartile range (IQR). The Shapiro–Wilk test was used to determine the normality of distributions. Mann–Whitney U test (or t-test, when appropriate) for continuous variables and chi-square test for categorical variables were used to assess differences in sociodemographic, clinical characteristics, and scores on the COPE and EQ-5D between NT1 and the CG. The association between coping strategies and quality of life was assessed using Spearman's rho correlation in the patient group. P-values were adjusted by the Benjamini–Hochberg false discovery rate (FDR) multiple testing correction^28^. Correlation coefficients whose magnitude is between 0.5 and 1 indicate variables that can be considered strongly correlated. Correlation coefficients whose magnitude is between 0.3 and 0.5 indicate variables that have a moderate correlation. Correlation coefficients whose magnitude is less than 0.3 have a weak correlation^29^. A *p* -value < 0.05 was considered significant. Statistical analysis was performed with Stata SE 14.2.

## Results

One hundred and twenty-two patients completed all the questionnaires and were matched for sex and age with 138 controls. Descriptive analyses of the two groups were reported in Table 1. Results showed that a higher percentage of NT1 patients reported low levels of education (23.8 vs. 10.1%, *p* = 0.03) and were less likely to be employed (51.6 vs. 68.1%). One hundred eleven patients were receiving pharmacotherapy; 60 participants were on monotherapy and 51 were on polytherapy (i.e., a combination of two or three drugs). Sodium oxybate (n = 75), stimulants (n = 66), and anti-cataplectics (n = 27) were among the prescribed drugs reported. Eleven patients reported that they were not currently receiving pharmacotherapy.

**Table 1:** Demographic and clinical features of NT1 patients versus controls.

	NT1 (n = 122)	CG (n = 138)	*p*
Age—years			0.119
Mean (SD)	37.5 (15.3)	39.5 (14.1)	
Median [IQR]	38 [22–46]	40 [25–48]	
Sex n (%)			0.365
Female	63 (51.6)	79 (57.3)	
Low level of education n (%)			**0.003**
Yes	29 (23.8)	14 (10.1)	
Not working n (%)			**0.007**
Yes	59 (48.4)	44 (31.9)	
Age of onset of symptoms—years			
Mean (SD)	20 (12.5)		
Median [IQR]	16 [12–25]		
Age at diagnosis—years			
Mean (SD)	27.9 (13.5)		
Median [IQR]	25 [17–36]		
Currently on pharmacotherapy N (%)			
Yes	111 (91.0)		
Monotherapy	60 (49)		
Polytherapy	51 (51)		
EQ5D			** < 0.001**
Mean (SD)	0.83 (0.16)	0.91 (0.10)	
Median [IQR]	0.84 [0.79–1]	1 [0.83–1]	

Bold values denote statistical significance at the *p* < 0.05 level.

*EQ5D* EuroQol-5D.

Patients with NT1 reported significantly lower levels of quality of life than CG; the mean EQ5D score for the narcolepsy group was 0.83 (SD = 0.16), while the mean score for the CG was 0.91 (SD = 0.10), with a statistically significant difference between the two groups (*p* < 0.001).

## Comparison of coping strategies in patients with NT1 and controls

Furthermore, patients with NT1 reported a different pattern of coping strategies compared to participants in the CG. Specifically, as shown in Table 2, patients reported significantly higher levels of behavioral and mental disengagements and lower levels of the following coping strategies: active coping, planning, use of instrumental social support, use of emotional social support, suppression of competing interests, positive reinterpretation and growth, and focusing on and venting of emotion.

**Table 2:** Differences in the levels of coping strategies between patients with narcolepsy and controls.

	Patients with NT1 (n = 122)	Control group (n = 138)	*p*
Active coping			** < 0.001**
Mean (SD)	6 (2.4)	7.4 (2.5)	
Median [IQR]	6 [4–8]	7 [6–9]	
Planning			** < 0.001**
Mean (SD)	6.1 (2.9)	7.8 (2.7)	
Median [IQR]	6 [4–8]	8 [6–10]	
Suppression of competing activities			**0.003**
Mean (SD)	4.6 (2.6)	5.6 (2.4)	
Median [IQR]	5 [3–6]	5 [4–7]	
Restraint			0.741
Mean (SD)	5.2 (2.6)	5.3 (2.5)	
Median [IQR]	5 [3–7]	5 [3–7]	
Use of instrumental social support			**0.020**
Mean (SD)	5.5 (3)	6.4 (3)	
Median [IQR]	6 [4–7]	7 [4–9]	
Use of emotional social support			**0.009**
Mean (SD)	4.5 (3.3)	5.5 (3.5)	
Median [IQR]	4 [2–7]	6 [3–8]	
Focusing on and venting of emotions			**0.009**
Mean (SD)	4.4 (2.9)	5.3 (2.7)	
Median [IQR]	4 [2–7]	5 [3–7]	
Positive reinterpretation and growth			** < 0.001**
Mean (SD)	6.5 (3.3)	7.9 (2.6)	
Median [IQR]	7 [4–9]	8 [6–10]	
Acceptance			0.734
Mean (SD)	6.6 (2.8)	6.5 (2.7)	
Median [IQR]	7 [4–9]	6 [4–8]	
Religion			0.464
Mean (SD)	2.5 (3.5)	2.4 (3.6)	
Median [IQR]	0.5 [0–4]	0 [0–4]	
Humor			0.234
Mean (SD)	3.1 (2.9)	3.5 (2.8)	
Median [IQR]	3 [1–5]	3 [1–5]	
Denial			0.259
Mean (SD)	1.7 (2.2)	1.3 (1.7)	
Median [IQR]	1 [0–3]	1 [0–2]	
Behavioral disengagement			**0.002**
Mean (SD)	2.5 (2.6)	1.5 (1.8)	
Median [IQR]	2 [0–4]	1 [0–3]	
Mental disengagement			**0.014**
Mean (SD)	4.2 (2.3)	3.5 (2.3)	
Median [IQR]	4 [3–6]	3 [1–5]	
Substance use			0.292
Mean (SD)	0.6 (1.6)	0.5 (1.5)	
Median [IQR]	0 [0–0]	0 [0–0]	

Bold values denote statistical significance at the *p* < 0.05 level.

## Correlation between coping strategies and quality of life in patients with NT1

The results of Spearman's rank correlations in patients with NT1 indicate that denial and behavioral disengagement were significantly and negatively associated with quality of life, indicating that higher levels of these coping strategies were associated with lower levels of quality of life (rho = − 0.33, adj-*p* < 0.001; rho = − 0.35, adj-*p* < 0.001; rho = − 0.29, adj-*p* = 0.006, respectively). Spearman rank correlation results are displayed in supplementary Table S2 (see Supplementary Material).

## Discussion

Our findings show that patients have significantly lower EQ5D scores compared to healthy controls (i.e., 0.83 vs. 0.91). Additional evidence has indicated that patients with narcolepsy experience a lower quality of life compared to controls^30–33^. Furthermore, our findings align with the mean EQ5D score (i.e., 0.85) documented in six studies investigating the quality of life among patients with narcolepsy employing the same assessment tool, as outlined in a recent systematic review^8^. Notably, evidence suggests that patients with narcolepsy may exhibit comparable levels of quality of life to those with epilepsy^34^ or even lower^35^, depending on the available data. Several factors might contribute to the reduced quality of life experienced by patients with narcolepsy. Firstly, the disorder's symptoms, such as excessive daytime sleepiness, cataplexy, sleep disturbances, and cognitive impairments, can significantly impair daily functioning and overall well-being. These symptoms often lead to difficulties in maintaining employment, social relationships, and engaging in recreational activities, thereby impacting various domains of life.

Concerning the primary objective, we observed that patients with NT1 exhibit distinct patterns in their use of coping strategies compared to controls. Specifically, patients with NT1 reported lower use of active coping, planning, and suppression of competing activities. Notably, previous evidence highlighted impairments in executive patients with NT1^36,37^, and since coping strategies often rely on multiple components of executive function (e.g., working memory, planning, sequencing, cognitive flexibility, and inhibitory control)^38–40^, it might be interesting for future research if impairments in executive functioning might have an impact on the adoption of adaptive coping strategies. Additionally, they sought less emotional and instrumental support, potentially due to stigma or feelings of shame surrounding their condition^41,42^. NT1 patients also employed less focusing on and venting of emotions, positive reinterpretation, and personal growth. The tendency to avoid or suppress emotions in people with NT1 may potentially stem from the fear of exacerbating emotional challenges associated with the condition, as well as the fear of triggering cataplectic attacks, which are known to be triggered by intense emotions^10^. Also, it could be hypothesized that in patients with NT1, the use of positive reinterpretation and growth as adaptive coping strategies may be hindered by depressive symptoms, which often co-occur in narcolepsy^11^ and prevent individuals from actively engaging in this coping strategy aimed at pursuing meaning, personal growth, or learning opportunities within the adversity they face.

On the other hand, patients with NT1 reported using mental and behavioral disengagement more frequently. Specifically, mental disengagement allows individuals to mentally distance themselves from the stressors, while behavioral disengagement is characterized by a passive and avoidant approach to dealing with problems or stressors. People who use mental and behavioral disengagement withdraw from the pressures and refrain from taking any action, as opposed to actively trying to address the problem. As previously mentioned, living with narcolepsy has a significant psychological and emotional toll, which may motivate the employment of mental and behavioral disengagement coping strategies as a way to regulate and reduce their emotional distress. Although these coping strategies may provide temporary emotional relief, it is important to note that relying excessively on them may inhibit long-term adaptation and adjustment to the disease^43^.

With respect to our second objective, our results showed that the use of specific coping strategies, namely denial and behavioral disengagement, was negatively associated with quality of life in this population. Denial is a coping strategy that entails downplaying or ignoring a problem. Importantly, when the stressor is represented by a chronic health condition such as NT1, denial may prevent individuals from seeking appropriate treatment or taking the necessary steps to manage their illness. Similarly, behavioral disengagement is a maladaptive strategy characterized by withdrawal and  avoidance, and entails reducing effort or involvement in coping efforts. These findings, which have never been examined in the literature among patients with NT1, are consistent with the available data and demonstrate that these approaches are typically ineffective when dealing with chronic health conditions^21–23,43,44^. For example, in a previous study on individuals with multiple sclerosis, it was found that avoidant coping strategies, such as denial and disengagement, are associated with negative outcomes such as decreased social engagement, impaired physical functioning, and increased depressive symptoms^45^. Moreover, according to a meta-analysis conducted with patients with breast cancer, avoidance and disengagement appear to be consistently ineffective and are associated with poorer psychological and physical functioning^44^.

Furthermore, the combination of these two coping strategies can potentially impede treatment compliance. Indeed, the treatment of NT1 requires lifestyle changes, including maintaining a regular sleep schedule and adhering to a medication plan^5^. However, individuals who engage in denial or behavioral disengagement may overlook the importance of these crucial lifestyle modifications and exhibit resistance to regularly taking prescribed medications. Even though these two strategies might serve as temporary coping mechanisms providing emotional relief, in the long run, they can hinder effective illness adaptation and symptom management, potentially exacerbating the challenges associated with the illness^43^.

Overall, our findings suggest that patients may benefit from interventions that encourage adaptive coping techniques, including active coping and the search for emotional and practical assistance, as well as those that discourage the use of disengagement and denial. Healthcare providers should collaborate closely with patients to develop a treatment plan that includes interventions intended to develop a broader and more effective repertoire of coping strategies. This recommendation is consistent with current guidelines for the management of narcolepsy that underscore the importance of a combination of pharmacological and psychological interventions^46^.

As a final remark, according to our results patients with NT1 reported lower levels of education and higher levels of unemployment. This is in line with previous evidence that highlights that narcolepsy was responsible for career curtailment at a productive age and for living with partial or complete disability^47,48^. However, the findings appear to be conflicting, as other existing evidence regarding educational and professional trajectories has indicated no significant impairment when compared to a control group^49^. Several research investigations have consistently indicated a correlation between socioeconomic status and quality of life^50^. Moreover, findings across various studies focusing on individuals with chronic health conditions consistently demonstrate that those with lower socioeconomic status tend to exhibit lower levels of quality of life^51^. Future studies assessing the impact of narcolepsy on quality of life should consider these variables. Additionally, support interventions for NT1 and academic and labor market policies should integrate strategies aimed at promoting higher levels of education and employment where feasible, thus addressing the socio-economic challenges faced by individuals with narcolepsy and potentially improving their overall well-being. Several limitations should be considered when interpreting the study results. The study was carried out in a single Narcolepsy Centre in Bologna, Italy, which can restrict the generalizability of the results to other settings or cultural contexts. The study relied exclusively on self-report measures to assess coping strategies and health-related quality of life. The study employed a cross-sectional design, limiting the ability to establish causal relationships between coping strategies and quality of life. Longitudinal studies are needed to examine the stability and variability of coping strategies and their association with quality of life in individuals with narcolepsy. Finally, including family members or companions of patients in the control group may introduce bias, thus potentially constraining the generalizability of the findings. This is primarily due to the potential for these individuals to share similar socioeconomic backgrounds, lifestyles, and environmental factors with the patients themselves.

### Supplementary Information

Supplementary Information.

## Data Availability

The datasets generated during and/or analyzed during the current study are available from the corresponding author on reasonable request.
