# Supplementary material for: Navigating narcolepsy: exploring coping strategies and their association with quality of life in patients with narcolepsy type 1
Source: Sci Rep. 2024 May 23;14:11837. doi: 10.1038/s41598-024-62698-5 (PMC11116444; doi:10.1038/s41598-024-62698-5)
Supplement: Supplementary file 1 — Supplementary Information. [file 41598_2024_62698_MOESM1_ESM.docx]

| **Supplementary Material table S1. Brief description of coping strategies measured by the COPE Inventory according to Carver’s framework** | |
| --- | --- |
| Active coping | The use of proactive, adaptive stress management strategies, such as seeking support or taking action to address the problem. |
| Planning | Coping with stress through problem-solving and proactive planning |
| Suppression of competing activities | Putting aside other activities, avoiding distractions, and letting other things slide to deal with the stressor. |
| Restraint | Waiting for the right moment to act, holding back, and avoiding acting prematurely |
| Seeking social support for instrumental reasons | Seeking advice, practical aid, resources, or information to manage stress. |
| Seeking social support for emotional reasons | Seeking emotional support,  Moral support, sympathy, or understanding from others to cope with stress |
| Focusing on and venting of emotions | Coping with stress by expressing negative emotions and seeking emotional release. |
| Positive reinterpretation and growth | The use of positive thinking and cognitive reappraisal to cope with stress. |
| Acceptance | Accepts the reality of a stressful and demanging situation |
| Religion | Inclination to rely on religion during times of stress. |
| Humor | Making fun of the situation, laughing about it |
| Denial | Denying the existence or significance of the stressor as a way to cope. |
| Behavioral disengagement | Avoiding or withdrawing from the stressor or situation as a way to cope. |
| Mental disengagement | Psychologically detaching oneself from the problem by avoiding thinking about it or by engaging in distracting activities. |
| Substance use | Using drugs or alcohol to cope with stress. |
|  |  |
|  |  |

**Supplementary Material table S2. Association between coping strategies and quality of life in patients with NT1 (N=122)**

|  | Spearman's rho correlations with EQ-5D | *p* | Adj-*p* |
| --- | --- | --- | --- |
| Active coping | 0.11 | .227 | .558 |
| Planning | 0.07 | .447 | .745 |
| Suppression of competing activities | -0.15 | .099 | .330 |
| Restraint | -0.06 | .531 | .779 |
| Use of instrumental social support | 0.03 | .779 | .898 |
| Use of emotional social support | -0.11 | .216 | .558 |
| Focusing on and venting of emotions | -0.19 | .041 | .153 |
| Positive reinterpretation and growth | 0.07 | .422 | .745 |
| Acceptance | 0.09 | .316 | .631 |
| Religion | -0.14 | .132 | .397 |
| Humor | 0.06 | .503 | .779 |
| Denial | -0.33 | <.001 | **.001** |
| Behavioral disengagement | -0.35 | <.001 | **.001** |
| Mental disengagement | -0.23 | .013 | .054 |
| Substance use | -0.01 | .919 | .933 |
| Note. EQ5D: Euro-Qol Group; Bold values denote statistical significance at the *p* < 0.05 level. | | | |
